# Effects of purified or plant-produced Cry proteins on *Drosophila melanogaster* (Diptera: Drosophilidae) larvae

**DOI:** 10.1038/s41598-017-10801-4

**Published:** 2017-09-11

**Authors:** Simone Haller, Jörg Romeis, Michael Meissle

**Affiliations:** Agroscope, Research Division Agroecology and Environment, Reckenholzstrasse 191, 8046 Zurich, Switzerland

## Abstract

Although genetically engineered crops producing insecticidal Cry proteins from *Bacillus thuringiensis* (*Bt*) are grown worldwide, few studies cover effects of *Bt* crops or Cry proteins on dipteran species in an agricultural context. We tested the toxicity of six purified Cry proteins and of *Bt* cotton and *Bt* maize tissue on *Drosophila melanogaster* (Diptera: Drosophilidae) as a surrogate for decomposing Diptera. ELISA confirmed the presence of Cry proteins in plant material, artificial diet, and fly larvae, and concentrations were estimated. Median concentrations in emerging adult flies were below the limit of detection. Bioactivity of purified Cry proteins in the diet was confirmed by sensitive species assays using *Heliothis virescens* (Lepidoptera: Noctuidae). Purified Cry1Ab, Cry1Ac, Cry1B, Cry1C, Cry1F, or Cry2Aa, or leaf material from stacked *Bt* cotton (Bollgard II producing Cry1Ac and Cry2Ab) or *Bt* maize (SmartStax producing Cry1A.105, Cry1Fa2, Cry2Ab2, Cry3Bb1, Cry34Ab1 and Cry35Ab1) had no consistent effects on *D. melanogaster* survival, developmental time, adult body mass or morphometrics. However, *D. melanogaster* showed longer developmental time and smaller wing size when fed with cotton leaves from plants infested with *H. virescens* caterpillars compared to flies fed with leaves from uninfested plants, while no such effects were obvious for maize.

## Introduction

Maize and cotton with resistance against pest Lepidoptera and/or Coleoptera are among the most important genetically engineered (GE) crops worldwide^1^. The insect resistance trait has been achieved by integrating one or several *cry* genes from *Bacillus thuringiensis* (*Bt*), a bacterium producing parasporal crystalline proteins with specific pesticidal activities^2^, into the crop genome. Different subspecies or strains of *Bt* produce specific Cry proteins with biological activity usually restricted to certain insect orders. So is the subspecies *kurstaki* and *aizawai* active against Lepidoptera, *tenebrionis* against Coleoptera and *israelensis* against Diptera. The narrow spectrum of activity observed for *Bt* has led to its use as a valuable pest control tool^3^ for many years mainly in integrated and organic production.

One concern with the use of insecticidal GE plants is that they may potentially harm non-target organisms fulfilling important ecosystem services^4, 5^. Certain Diptera (flies and midges) provide important ecological functions, such as decomposition^6^, predation^7^, pollination^8^, and parasitism^9^. Despite their ecological importance, only few studies with Diptera and GE plant material or purified Cry proteins are available. One example is the study by Knecht and Nentwig^10^ who reported no adverse effects when larvae of *Drosophila melanogaster* (Diptera: Drosophilidae) and *Megaselia scalaris* (Diptera: Phoridae) were fed for multiple generations with a diet that contained decaying *Bt* maize leaves (expressing either Cry1Ab or Cry3Bb1).

Van Frankenhuyzen^11^ reviewed available evidence on the activity spectra of Cry and Cyt proteins from *Bt*. Dipteran species with socio-economic importance, i.e. disease vectors like *Aedes aegypti*, *Anopheles stephensi*, *Culex pipiens*, and *C. quinquefasciatus* (all Diptera: Culicidae) were most frequently tested. Different protein families, such as Cry1, Cry2, Cry4, Cry11, Cyt1, and Cyt2, were observed to have activity against Diptera^11^. Of those, however, only Cry1 and Cry2 are of practical relevance in an agricultural context, because they are produced by today’s *Bt* crops and provide resistance against pest Lepidoptera. In contrast, Cry3 proteins, which are produced in *Bt* maize resistant to chrysomelid beetles, were reported to have no dipteran activity^11^.

In addition to the potential for individual Cry proteins to affect Diptera, the stacking of multiple Cry proteins into plants has raised concerns that Cry proteins may interact synergistically when simultaneously expressed in plants, potentially leading to unexpected non-target effects^3, 12^. However, no data on combinatorial effects of multiple Cry proteins in stacked plants on Diptera are available.

In this study, we focused on *D. melanogaster* as a representative for the decomposer Diptera. The aims of the study were to evaluate potential effects of a) plant material from stacked *Bt* plants (cotton and maize) expressing a combination of Cry proteins, and b) a range of different purified Cry1 and Cry2 proteins on *D. melanogaster*. For the assays with plant material, we compared the effects of leaves from *Heliothis virescens* caterpillar-infested plants with those from uninfested plants on *D. melanogaster*, because particularly cotton is known to have a natural plant defence system based on terpenoid production that is induced by caterpillar damage^13, 14^.

## Material and Methods

### Plant material

Plants were cultivated in a climate chamber with daily fluctuating light intensity (16.5 h light), temperature (22 to 28 °C) and humidity (45 to 85%) to simulate natural climate conditions. We used the GE cotton event MON15985 (Bollgard II, Monsanto Company) expressing *cry1Ac* and *cry2Ab* and the near-isoline control cultivar Sure-Grow 125. The second GE plant used was maize event MON 89034 × TC1507 × MON 88017 × DAS-59122-7 (SmartStax, Monsanto Company and Dow AgroSciences LLC) expressing *cry1A.105*, *cry1Fa2*, *cry2Ab2*, *cry3Bb1*, *cry34Ab1*, and *cry35Ab1*. The conventional cultivar EXP258 served as a closely related control. All seeds were supplied by Monsanto Company.

Cotton and maize plants were grown individually in 3l plastic pots containing heat-sterilized humus-rich soil and 15 g slow release fertilizer (15% N, 7% P_2_O_5_, 15% K_2_O, Manna, Wilhelm Haug, Ammerbuch, Germany). Plants were watered daily and fertilized weekly using approximately 100 ml of a 1% solution of liquid fertilizer (10% N, 6% P_2_O_5_, 6% K_2_O, Manna, Wilhelm Haug). After three weeks, half of the plants were infested with a single second or third instar of *Heliothis virescens* (Lepidoptera: Noctuidae) to trigger natural plant defence induction^14, 15^. The larvae were placed on the youngest fully developed leaf and enclosed using a gauze bag. After one week all newly developed leaves were harvested, pooled for all plants of each treatment, and stored at −80 °C. The plant material was lyophilized and milled to a fine powder using a coffee mill (Mio Star, Migros, Switzerland).

### Insects

Feeding experiments were conducted with a strain of *D. melanogaster* that had been used in earlier studies in our laboratory^16^. The flies were initially provided by W. J. Gehring (University of Basel, Switzerland) and cultured at Agroscope for several generations on an artificial diet according to Haller *et al*.^16^. Freshly laid eggs (<4 h old) of *D. melanogaster* were used in the feeding experiments.

A strain of *H. virescens* was provided by Bayer CropScience (Monheim, Germany) and reared on an artificial diet modified after Teakle and Jensen^17^ at Agroscope. Second or third instars were used to infest maize and cotton plants and neonates were used for sensitive insect bioassays. Both insect cultures were maintained in a climate chamber at 25 ± 1 °C with 70 ± 10% humidity and a photoperiod of 16 hours.

### Insecticidal compounds

Insecticidal compounds used in this study included a synthetic form of the mineral pesticide cryolite (Na_3_AlF_6_, sodium hexafluoroaluminate) (purity: ≥97.0%) (Sigma-Aldrich, Buchs, Switzerland) and the lyophilized *Bt* proteins Cry1Ab, Cry1Ac, Cry1B, Cry1C, Cry1F, and Cry2Aa (purity 94–96%), which were provided by Marianne Pusztai-Carey (Case Western Reserve University, Cleveland, USA). Cryolite and Cry proteins are gut toxicants that require ingestion for activity.

### Bioassays with *Bt* plant material

Bioassays were conducted with leaf material from *Bt* maize (SmartStax) and *Bt* cotton (Bollgard II) that had either been caterpillar-infested or remained uninfested. Plant material from non-*Bt* maize and non-*Bt* cotton, either caterpillar-infested or uninfested, were used as controls. The bioassays followed the “single-fly” protocol by Haller *et al*.^16^ with the following modification: the standard *D. melanogaster* diet was prepared with the amount of solid ingredients reduced by one third. Once the diet cooled down below 55 °C, it was poured into plastic boxes and the milled plant material was added immediately with steady stirring. A ratio of 1 to 3 (plant material to artificial diet, based on the dry components of the diet) was used since it was found to allow normal development of the flies in preliminary experiments with control plant material. In addition, a diet-only (negative) control and two positive controls containing 0.04 and 0.004% (w/w) cryolite in artificial diet were used.

The artificial diet including the test material was distributed in 96-well microtiter plates. After one freshly laid egg (<4 h old) of *D. melanogaster* was added to each test well, the plates were sealed, stored in the climate chamber (25 ± 1 °C, 70 ± 10% humidity, photoperiod 16 hours), and checked every day until all adults had emerged. The following parameters were recorded: survival (emergence of an adult fly), developmental time, adult dry weight, and wing size^16^.

Three independent trials were conducted, each with 24 replications per treatment. Wells in which the diet dried out or which were contaminated with fungi were excluded from analyses, resulting in a total of 59 to 71 replications per treatment.

### Bioassays with purified Cry proteins

Bioassays conducted with purified Cry proteins followed the single-fly protocol^16^.

All Cry proteins were tested at a concentration of 100 µg/g fresh artificial diet (0.01%), which is in the upper part of the range of the concentrations of Cry proteins in commercialized *Bt* plants (see Supplemental Fig. S2). The bioactivity of the Cry proteins in the artificial diet was determined by a “sensitive-insect” bioassay (see below). Cryolite was applied as a positive control compound at 0.04 and 0.004% (w/w) and a pure diet treatment served as the negative control. The following parameters were recorded when all adults had emerged: survival (emergence of an adult fly), developmental time, adult dry weight and wing size.

Four independent trials were conducted, each with 21 replications per treatment. An exception was the negative control treatment, which was conducted with 42 replications^18^. One of the trials, however, had to be discarded because the control mortality exceeded 30%; a quality criterion established in Haller *et al*.^16^. Additionally, wells in which the diet dried out or which were contaminated with fungi, were excluded from analyses, resulting in a total of 51 to 61 replications per treatment and 110 replications for the negative control treatment that could be used for analyses.

### Biological activity of purified Cry proteins in *D. melanogaster* diet

The bioactivity of the purified Cry proteins was assessed in fresh and 5 day old *D. melanogaster* diet. This 5 day period reflects the time of active feeding of the *D. melanogaster* larvae and thus exposure to the Cry proteins until they reached the pupal stage. After the diet including purified Cry proteins was prepared for the *D. melanogaster* bioassay, a portion of the surplus of each treatment was collected for Cry protein measurements. Another portion of the surplus of each treatment was incubated for 5 days under the same conditions as the bioassay and then collected. Fresh and 5 day old diet samples from each trial were stored at −80 °C until used for analysis.

Bioactivity was assessed using the test species *H. virescens*, which is known to be sensitive to Cry1A, Cry1F, and Cry2A proteins. The diet samples were lyophilized (the weight of the lyophilized diet was on average 15.9% of the fresh diet) and milled for 2 minutes with one 3 mm tungsten carbide ball at a frequency of 30 Hz using a mixer mill (Retsch MM300, Retsch GmbH, Haan, Germany). The milled *D. melanogaster* diet was subsequently mixed with 2.5 g Stonefly *Heliothis* diet powder (Ward’s Science, Rochester, NY, USA). The amount of milled *D. melanogaster* diet added depended on the expected LC_50_ values of the different Cry proteins for *H. virescens* according to the literature where available: Cry1Ab, Cry1Ac and Cry2Aa: 10 mg (high sensitivity); Cry1F: 100 mg (medium sensitivity); Cry1B and Cry1C: 1000 mg (low sensitivity). Pure Stonefly *Heliothis* diet and Stonefly *Heliothis* diet containing 10 mg, 100 mg, and 1000 mg of *D. melanogaster* diet without Cry proteins served as negative control treatments. Subsequently, the diet powder was mixed with 400 µl white wine vinegar (Spar, Switzerland) and 7.1 ml tap water. The diet of each treatment was transferred into a small plastic box (50 mm × 30 mm × 15 mm) and cut into 20 cubes, using a scalpel. The cubes were transferred to 128-well plastic bioassay trays (Bio-Serv, Flemington, NJ, USA). With a fine brush, one *H. virescens* neonate (<24 h old) was added to each well and the wells were sealed with 16 cell tray covers (Bio-Serv). After incubation for one week at 25 ± 1 °C, 70 ± 10% humidity, and a 16 h photoperiod, the larval survival rate was determined and the larval fresh weight was recorded with a Mettler AT261 DeltaRange^®^ balance (Mettler Toledo, Greifensee, Switzerland).

Because of the high number of treatments (4 controls and 6 Cry proteins, each with fresh and 5 day old diet), the sensitive insect assay was performed in two parts: Part A included Cry1B, Cry1C, and Cry1F with the 1000 mg and 100 mg control treatments. Part B included Cry1Ab, Cry1Ac, and Cry2Aa with the 10 mg control treatment. Each treatment was tested twice (2 trials), each with 20 *H. virescens* larvae per treatment.

Wells in which the diet dried out during the bioassay or which were contaminated with fungi, were excluded from the analyses, resulting in a total number of 34 to 40 larvae per treatment.

### Quantification of Cry proteins

The Cry protein concentrations in leaf powder, artificial diet, and *D. melanogaster* adults and larvae were measured with double-antibody sandwich enzyme-linked immunosorbent assays (DAS-ELISA) using 96-well PathoScreen kits (Agdia, Elkhart, USA). From the leaf tissue used for the plant assays, five subsamples for Cry protein analysis were taken from each of the lyophilized and milled caterpillar-infested or uninfested *Bt* maize and *Bt* cotton leaf tissues. In addition, five subsamples were taken from each of the milled fresh and 5 day old *D. melanogaster* diets treated with purified Cry1Ab, Cry1Ac, Cry1B, Cry1C, Cry1F and Cry2Aa used for the biological activity assays. Three subsamples were also taken from the respective non-*Bt* leaf powders and from the control diets to test for unspecific binding of the antibodies in the ELISA kits.

To quantify Cry proteins in *D. melanogaster* adults and larvae, the offspring of 6 pairs of flies was reared to pupae, either on diet containing *Bt* cotton (ratio 1:3) or on diet containing *Bt* maize (ratio 1:3). The offspring of each pair of flies remained in the same container. Pupae were separated from the artificial diet to avoid ingestion of Cry protein after emergence. Adults were weighed and stored at −80 °C. Five samples of adults, each containing 12–23 pooled individuals were collected. Additional pairs of flies were set up in a similar way and late larvae were collected before pupation. Larvae were washed with tap water, dried on tissue paper, weighed, and stored at −80 °C. Five samples of larvae, each consisting of 7 to 10 pooled individuals, were collected.

All samples were lyophilized before ELISA measurements. Commercial kits were available for Cry1Ab/Cry1Ac, Cry1F, Cry2A, Cry3Bb1, and Cry34Ab1. Detection of Cry1A.105 was possible with the kit for Cry1Ab/Cry1Ac albeit with lower sensitivity. Protein concentrations of Cry1B and Cry1C were not measured since there were no kits available. Proteins were extracted in 500 µl extraction buffer; phosphate-buffered saline with 0.55% Tween-20 (PBST). A 3 mm tungsten carbide ball was added to each tube with sample and buffer and samples were macerated for 2 min at 25 Hz in a mixer mill (Retsch MM300). After centrifugation for 5 min at 13000 × g, the supernatant was collected and centrifuged again at the same conditions. According to the estimated Cry protein concentrations, the supernatant of each sample was diluted with extraction buffer. ELISA plates were loaded with enzyme conjugate and sample according to the instructions provided with the kits. All detectable Cry proteins were analysed in each sample. Plates were incubated at 4 °C overnight and washed six times with wash buffer, i.e. PBST containing 0.05% Tween-20. The TMB substrate solution from the kit was added to the plates, incubated for 20 min at room temperature, and the optical density (OD) was measured at 620 nm light wave length using a SpectrafluorPlus plate reader (Tecan, Männedorf, Switzerland). To quantitate Cry proteins in samples, standard curves were constructed using purified proteins diluted in extraction buffer. Samples of *D. melanogaster* diet with purified Cry proteins were measured against standard curves of the same proteins provided by M. Pusztai-Carey. Plant and *D. melanogaster* samples were measured against standard curves of certified purified proteins provided by Monsanto (Cry1A.105, Cry1Ac, Cry2Ab, and Cry3Bb1) and Dow AgroSciences (Cry1F, Cry34Ab1). Concentrations of standards ranged between 1.25 and 80 ng protein/ml for Cry1A.105, between 0.31 and 20 ng protein/ml for Cry1Ab, Cry1Ac, Cry1F, Cry2Aa, Cry2Ab and Cry3Bb1, and between 0.078 and 5 ng protein/ml for Cry34Ab1. The Cry protein concentration in samples (µg Cry protein per g dry weight) was based on regression analysis of OD values and standard curve concentrations using a single rectangular, two parameter hyperbola model (Sigma-Plot 13, Systat Software). The limit of detection (LOD) was determined for each Cry protein by calculating the concentration based on 3 times the standard deviation of all blank values obtained for 10 ELISA plates of the same batch of kits.

### Determination of terpenoids in cotton

Natural plant defence, particularly known for cotton^13, 14^, includes the inducible production of terpenoids, which can be measured by high-performance liquid chromatography (HPLC). Twenty subsamples of 8 to 10 mg were taken from control and *Bt-*cotton leaf powder, both caterpillar-infested and uninfested. The terpenoid content (gossypol, hemigossypolone and heliocides 1 and 4) was analyzed using the protocol and machinery described by Hagenbucher *et al*.^15^.

To each subsample, 1 ml of extraction buffer, acetonitrile:H_2_O:orthophosphoric acid (acetonitrile: ≥99.0% Scharlau, Sentmenat, Spain; phosphoricacid: ≥85% Sigma-Aldrich, Buchs, Switzerland) in ratio of 80:20:0.1, was added and ultra-sonicated for 3 min at room temperature. After 3 min of centrifugation at 8000 × g, 300 to 400 µl of the extract was directly transferred into glass vials for separation and detection on a liquid chromatography system (Agilent 1260 Infinity, Agilent Technologies, Germany) equipped with a Varian Polaris Amide C-18 column (150 × 2.0 mm, 3 µm) and a precolumn (C18, 4 × 3.0 mm, Supelco Security Guard System) at a temperature of 40 °C. All substances to be analyzed in the plant extracts were baseline separated and detected with a single wavelength absorbance detector at 272 nm.

### Statistical analysis

All statistical analyses were conducted using the software R (version 3.0.2). For the *D. melanogaster* bioassays, the effect of treatments on survival were tested with a generalized linear model applying a binominal distribution and a Chi-square test for treatment and trial effects. Data for developmental time, female and male dry weight, and wing size were analyzed using a generalized linear model applying a quasipoisson distribution and an F-test for treatment and trial effects. If significant, treatment means were separated using Tukey’s test in the assay with *Bt* plant material. In the assay with purified Cry proteins, Dunnett’s test was used to compare treatment means to the negative control. For statistical analyses, the treatments were divided into three groups for the plant material bioassay: (1) the two cryolite treatments and the negative control, (2) cotton treatments, and (3) maize treatments. For the bioassay with purified Cry proteins, two groups of treatments were analyzed separately: (1) the two cryolite treatments were compared with the negative control, and (2) the Cry protein treatments were compared with the negative control. Data for developmental time, dry weight, and wing size were included in the analyses only for treatments with survival ≥ 20%^16^. If a significant treatment × trial interaction for any measurement endpoint was observed, the statistical analyses for that endpoint were repeated for each trial separately. Differences in the bioactivity assay with *H. virescens* were concluded from non-overlapping 95% confidence intervals. The ELISA data from the *D. melanogaster* diet containing Cry proteins were analyzed for differences among the fresh diet treatments and among the 5 day old treatments using Kruskal Wallis tests and for differences between fresh and old diet for each Cry protein separately using Mann-Whitney-Wilcoxon tests. The ELISA data from the plant material were analyzed for differences between infested and uninfested cotton or maize for each single Cry protein using Mann-Whitney-Wilcoxon tests.

## Results

### Bioassays with *Bt* plant material

**Figure 1:**
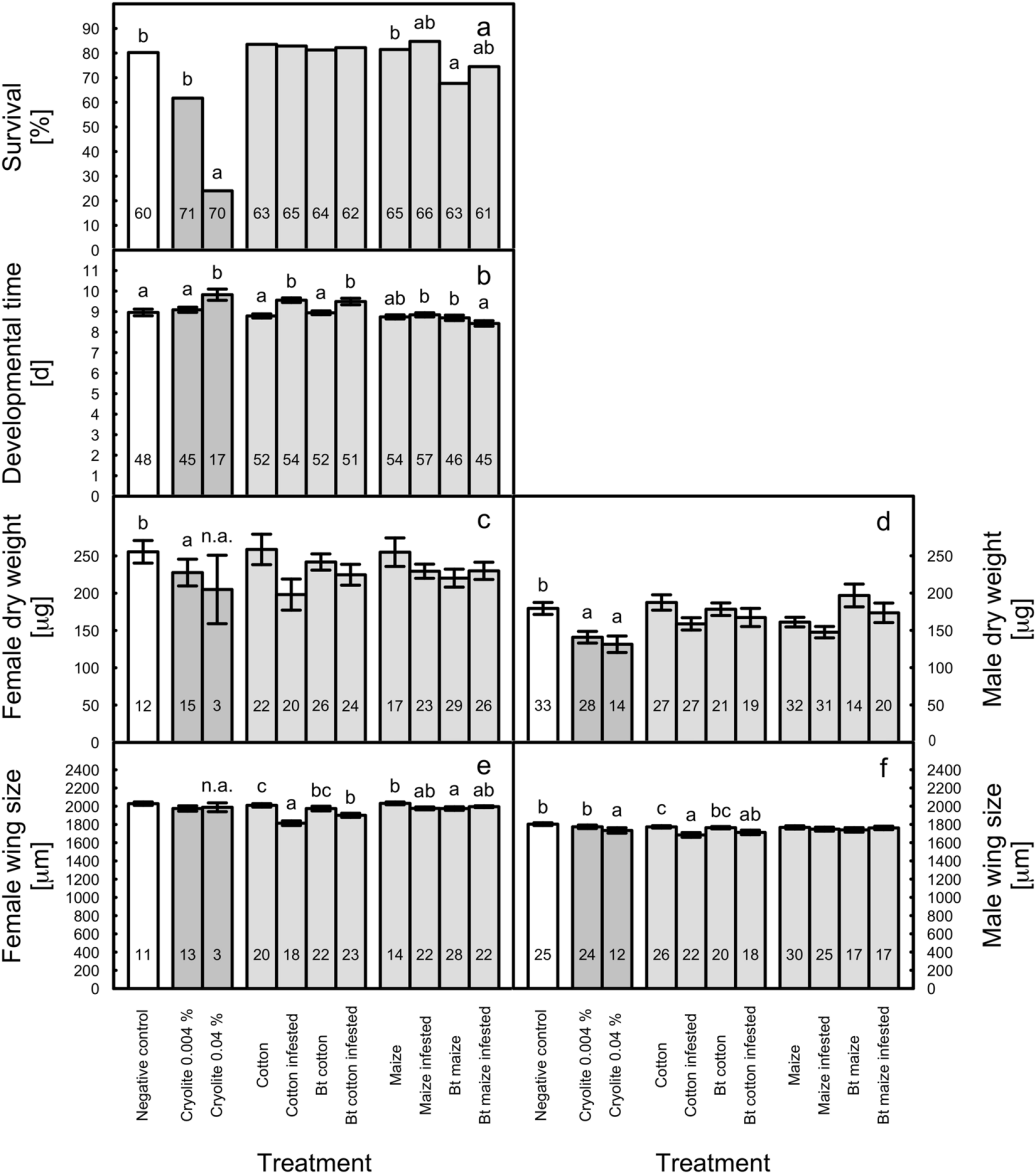
Effects of *Bt* and non-*Bt* cotton and maize, either caterpillar-infested or not infested, on (**a**) survival, (**b**) developmental time, (**c**) female dry weight, (**d**) male dry weight, (**e**) female wing size, and (**f**) male wing size of *D*. *melanogaster*. Cryolite at a concentration of 0.04% (w/w) and 0.004% (w/w) served as positive controls. The given means ± SE represent pooled values from three trials; the total numbers of replicates are indicated within the bars. Letters above bars indicate a significant difference between the treatments according to the generalized linear model using Chi-square statistics for the survival data and F statistics for the sublethal measurement endpoints. If significant, treatments were separated using Tukey’s test. Statistical analyses were conducted for three groups separately: (1) negative control and the cryolite treatments, (2) cotton treatments, (3) maize treatments; n.a. indicates that data were not included in the analyses because of a low number of surviving individuals.

When *Bt* or non-*Bt* cotton, either caterpillar-infested or uninfested, was mixed into the artificial diet for *D. melanogaster*, no difference in survival was detected across all trials (Fig. 1a). No significant difference was also observed when comparing the developmental time of *D. melanogaster* between *Bt* and non-*Bt* cotton leaf material, either caterpillar-infested or uninfested (Fig. 1b). Developmental time was increased by 8.1% (0.77 days) for *D. melanogaster* fed with infested non-*Bt* cotton and by 5.8% (0.55 days) for *D. melanogaster* fed with infested *Bt* cotton leaves compared to the respective non-infested leaves. Because the trial × treatment interaction for developmental time was significant, statistical analyses of this endpoint were conducted for each trial separately. In one of the three trials (trial 3), larvae fed with caterpillar-infested *Bt* cotton had a shorter developmental time than those grown on infested non-*Bt* cotton. In trials 1 and 2 no significant differences between the *Bt* and non-*Bt* treatments were observed (Supplemental Tables S1, S2). While both female and male dry weight did not differ significantly among the treatments (Fig. 1c,d), the wing size of both sexes did (Fig. 1e,f). Female wing size was reduced significantly when larvae were fed with infested *Bt* or non-*Bt* cotton material compared to uninfested non-*Bt* cotton (Fig. 1e). The wing size of females grown on uninfested *Bt* cotton did not differ from that of females on uninfested non-*Bt* cotton. However, infested non-*Bt* cotton resulted in lower female wing size than infested *Bt* cotton. Analysis of individual trials revealed that the latter observed difference can be attributed to one trial (trial 3), namely the one for which also a longer developmental time was observed (Supplemental Tables S1, S2). Similarly, male wing size was reduced significantly when *D. melanogaster* was reared on caterpillar-infested *Bt* or non-*Bt* cotton leaf material compared to uninfested non-*Bt* leaf material (Fig. 1f). No significant difference in male wing size was observed for flies grown on *Bt* cotton material compared with non-*Bt* material, either caterpillar-infested or uninfested. Analyses of individual trials, however, revealed that similar to females, males on infested non-*Bt* cotton had a lower wing size than males on infested *Bt* cotton in trial 3 (Supplemental Tables S1, S2).

For maize, the larvae on uninfested *Bt* maize had overall a 17% lower survival than the larvae on uninfested non-*Bt* maize (Fig. 1a). Because there was a significant trial × treatment interaction for survival, individual trials were analysed for this endpoint. Those analyses revealed that the effect of *Bt* maize on survival was only observed in trial 1 (Supplemental Tables S1, S2). The developmental time on infested *Bt* maize was significantly shorter compared to uninfested *Bt* maize and infested non-*Bt* maize (Fig. 1b). No statistical difference for developmental time was observed among the treatments with uninfested non-*Bt* maize, infested non-*Bt* maize, and uninfested *Bt* maize. Analyses of individual trials revealed that in trial 1 larvae developed faster in the uninfested non-*Bt* treatment than in the uninfested *Bt* treatment (Supplemental Tables S1, S2). No differences among treatments were observed for female and male dry weight (Fig. 1c,d). Female wing size of flies reared on uninfested *Bt* maize showed a 2.3% lower wing size compared to the uninfested non-*Bt* maize (Fig. 1e). Male wing size did not differ among treatments (Fig. 1f).

Cryolite at a concentration of 0.04% significantly reduced survival compared to the diet-only control (Fig. 1a). No significant effect on survival was observed for the lower (0.004%) cryolite concentration. Developmental time was significantly longer in the cryolite 0.04%, but not in the 0.004% treatment compared to the diet-only control (Fig. 1b). Female dry weight was reduced in the cryolite 0.004% treatment, while the low number of replicates (3) did not allow statistical analyses for 0.04% cryolite (Fig. 1c). Male dry weight was reduced in both cryolite treatments compared to the diet-only control (Fig. 1d). Considering female wing size, no treatment effect was detected in the 0.004% cryolite treatment, while no analysis was possible for 0.04% cryolite (Fig. 1e). Male wing size was reduced in the cryolite 0.04% treatment, but not in the cryolite 0.004% treatment compared to the diet-only control (Fig. 1f). Significant differences among the trials were observed for developmental time, female dry weight, male dry weight, and male wing size. No significant treatment × trial interactions were reported (Supplemental Table S2).

### Bioassays with purified Cry proteins

**Figure 2:**
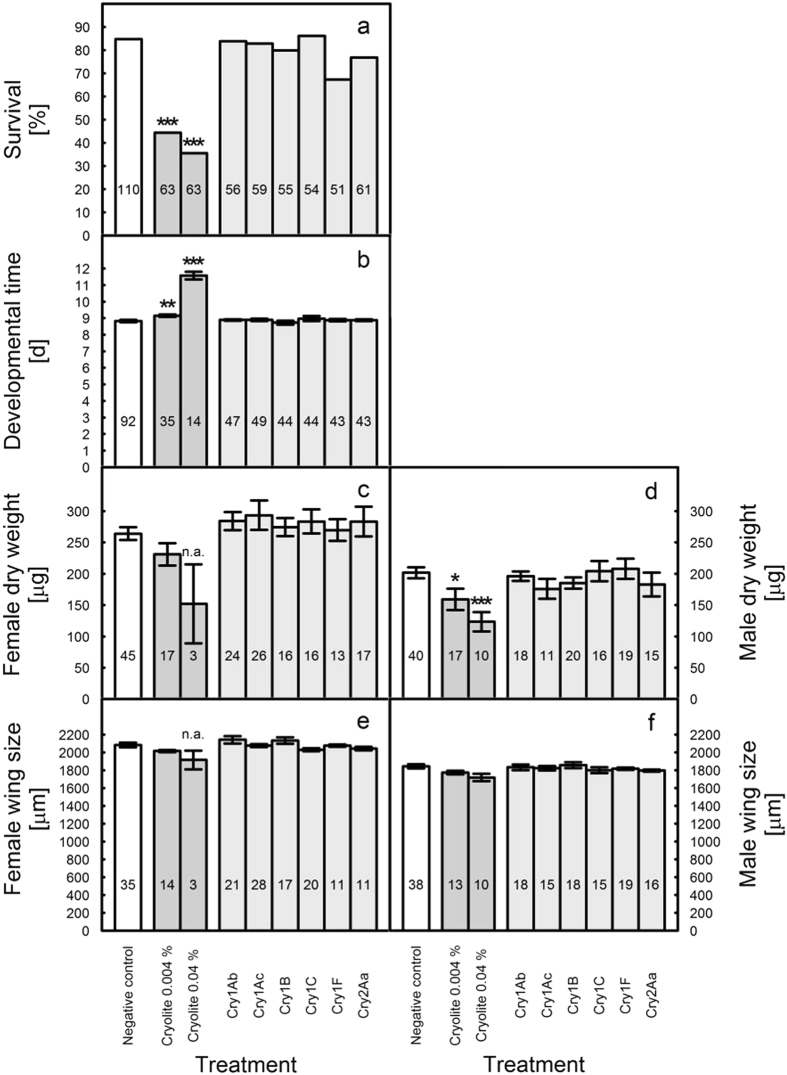
Effects of different Cry proteins (at 0.01%; w/w) on (**a**) survival, (**b**) developmental time, (**c**) female dry weight, (**d**) male dry weight, (**e**) female wing size, and (f) male wing size of *D*. *melanogaster*. Cryolite at a concentration of 0.04% (w/w) and 0.004% (w/w) served as positive controls. The given means ± SE represent pooled values from three trials; the total numbers of replicates are indicated within the bars. Asterisks (*p < 0.05, **p < 0.01, ***p < 0.005) above bars indicate a significant difference between the treatments according to the generalized linear model using Chi-square statistics for the survival data and F statistics for the sublethal measurement endpoints. If significant, treatments were separated from the negative control using Dunnett’s test. Statistical analyses were conducted for two groups separately: (1) the negative control compared with the two cryolite treatments and (2) the negative control compared with the *Bt* proteins; n.a. indicates that data were not included in the analyses because of a low number of surviving individuals.

In the overall analysis, no significant effects on survival and sublethal parameters of *D. melanogaster* were observed in the different treatments with Cry proteins (Fig. 2a–f). However, a significant trial × treatment interaction was found for developmental time, dry weight females, and dry weight males. Those endpoints were thus further analyzed for each trial. This evaluation revealed that in trial 1, larvae in the Cry1B treatment had a shorter developmental time and females in the Cry1Ac treatment a higher dry weight than those in the negative control treatment. For dry weight males, none of the individual trials was significant (Supplemental Tables S3, S4). Cryolite at both concentrations (0.04% and 0.004%) reduced survival, prolonged the developmental time, and reduced male dry weight (Fig. 2a,b,d). For male dry weight, analyses of individual trials revealed that a significant weight reduction compared to the negative control was only evident in the 0.004% cryolite treatment of trial 2 and in the 0.04% cryolite treatment of trial 3 (Supplemental Tables S3, S4). No effect on female dry weight was observed in the 0.004% cryolite treatment, while no analysis was possible in the 0.04% treatment due to a low number of surviving individuals (Fig. 2c). Wing size of males and females was not affected significantly in any treatment (Fig. 2e,f). For male wing size, however, the trial × treatment interaction was significant. In trial 3, males in the 0.04% cryolite treatment showed lower wing size than those in the negative control treatment (Supplemental Tables S3, S4).

### Biological activity of Cry proteins in *D. melanogaster* diet

The results from the sensitivity assay with *H. virescens* demonstrated that the Cry proteins, which were incorporated into the *D. melanogaster* diet, were bioactive (Table 1). Reduced survival of *H. virescens* was observed in both trials of the assay when neonates were fed with fresh artificial *D. melanogaster* diet containing Cry1Ac, Cry1F, and Cry2Aa, and in one trial when fed diet containing Cry1Ab. No effect on survival was observed in the Cry1B and Cry1C treatments. A reduction in weight increase was observed for Cry1Ab, Cry1Ac, and Cry2Aa in both trials of the assay and for Cry1B and Cry1C in one trial. Weight was not analyzed for Cry1F because of a low number of surviving individuals in the fresh diet treatment.

**Table 1:** Effects of *Drosophila melanogaster* diet containing different Cry proteins on survival and increase in fresh weight of *Heliothis virescens*. Artificial *Heliothis* diet containing the same amount of untreated *D. melanogaster* diet, which was also applied in the respective Cry treatments, served as negative controls. “yes” indicates lower survival or fresh weight of *H. virescens* in the Cry treatment *vs*. the control, either for fresh diet or for diet aged for 5 days, based on non-overlapping 95% confidence intervals in both experimental trials, “no” indicates overlapping 95% confidence intervals in both trials, “(yes)” indicates a significant effect in only one of the trials. For Cry1F, only 8 individuals survived in the fresh diet treatment, thus weight increase was not analyzed. Sample size per treatment and trial was 20. For more details see Supplemental Table S5.

	Fresh *Bt* diet vs. control		Aged *Bt* diet vs. control	
	Survival	Weight increase	Survival	Weight increase
Cry1Ab	(yes)	yes	no	no
Cry1Ac	yes	yes	no	yes
Cry1B	no	(yes)	no	(yes)
Cry1C	no	(yes)	no	(yes)
Cry1F	yes	n.a.	no	yes
Cry2Aa	yes	yes	yes	yes

When diet was incubated for 5 days, reduced survival of *H. virescens* was observed only for Cry2Aa. In contrast, reduced weight increase was evident for Cry1Ac, Cry1F, and Cry2Aa in both trials and for Cry1B and Cry1C in one trial. Thus bioactivity was confirmed also for aged diet based on weight reduction, except for Cry1Ab.

The detailed results from the individual trials are provided in the Supplemental Table S5.

### Cry protein analysis

Cry protein was detected in all analyzed *D. melanogaster* diets treated with Cry proteins (Supplemental Fig. 1), whereas in the control diets, no Cry protein was detected (data not shown). Measured Cry protein concentrations ranged from 192 (Cry1Ab) to 491 µg/g dry weight (Cry1F) in the freshly prepared *D. melanogaster* diet treatments and decreased over time for all Cry proteins. After 5 days, the diet contained approximately 24% of the initial Cry protein concentration for Cry1Ab, 16% for Cry1F, 26% for Cry1Ac, and 68% for Cry2Aa.

In *Bt* cotton samples, the Cry2Ab concentration was 111 µg/g, 47 times higher than the Cry1Ac concentration (2.0 µg/g). In *Bt* maize, the Cry3Bb1 concentration was the highest (81 µg/g) and Cry1Fa2 and Cry34Ab1 the lowest measured concentrations (10 µg/g). Caterpillar-infested and uninfested *Bt* plant material showed similar Cry protein concentrations, but concentrations of Cry1Ac in cotton and Cry1Fa2 and Cry34Ab in maize were higher (Supplemental Fig. 2). Control (non-*Bt*) plant samples were generally below the LOD with the exception of Cry2Ab in maize. The Cry2Ab concentration in control samples was, however, below 0.2% of the values measured in *Bt* maize.

*D. melanogaster* larvae, reared on diet with *Bt* cotton, contained no measurable Cry1Ac, but Cry2Ab was determined at 0.1 µg/g, which represents 0.1% of the Cry2Ab in cotton leaves. In larvae reared on diet with *Bt* maize, all Cry proteins were detected and concentrations ranged from 0.05 µg/g Cry2Ab (0.1% of maize leaves) to 0.24 µg/g Cry34Ab1 (2.4%) and 0.56 µg/g Cry1A.105 (1.5%). In adult flies, Cry protein concentrations were below the limit of detection for all samples except 3 samples out of 8 for Cry34Ab1 in the maize treatment (Supplemental Table S6).

### HPLC analyses of terpenoids in plant material

Terpenoid concentrations were increased in the leaves of the caterpillar-infested cotton plants compared with the uninfested plants, whereas *Bt* and non-*Bt* plants revealed similar concentrations independent of infestation (Table 2). The hemigossypolone value of the infested cotton was more than two- to three-fold compared to the uninfested plants for *Bt* and non-*Bt* cotton, respectively. The gossypol concentrations in the infested and non-infested *Bt* cotton were about twice the values measured in the uninfested plants. The heliocides H1/4 concentration in *Bt* cotton was also more than double in the infested plants compared to the uninfested ones. The biggest difference was observed in the non-*Bt* cotton plants, where the heliocides H1/4 value was around the five-fold level of the concentration measured in the uninfested plants.

**Table 2:** Terpenoid concentration (ng/mg DW) in pulverized leaves of *Bt* and non-*Bt* cotton (Bollgard II), which were used for the bioassays with *Drosophila melanogaster*. “Infested” indicates that the plants were infested with one *Heliothis virescens* larva for 7 days prior to the harvest of the leaf material. Values are means ± SE; n = 20 subsamples per treatment.

Plant material	Hemigossypolone [ng/mg DW]	Gossypol [ng/mg DW]	Heliocide H1/4 [ng/mg DW]
*Bt* cotton infested	6736.46 ± 250.72	317.18 ± 11.25	3077.39 ± 117.20
*Bt* cotton uninfested	3191.26 ± 119.33	173.93 ± 6.37	1381.59 ± 55.62
Non-*Bt* cotton infested	10301.83 ± 210.70	399.88 ± 28.64	6965.24 ± 158.74
Non-*Bt* cotton uninfested	3375.02 ± 104.00	216.25 ± 7.13	1018.61 ± 28.25

## Discussion

### Effects of Cry proteins on *D.melanogaster*

The bioassays with *D. melanogaster* did not reveal consistent effects of the tested *Bt* maize and *Bt* cotton materials or Cry proteins on lethal or sublethal measurement endpoints in comparison with the corresponding non-*Bt* diets. The experiment with *Bt* and non-*Bt* plant material was conducted in three independent trials. The outcome of the three trials was in parts variable, which is evident by significant trial effects and trial × treatment interactions. No overall difference among the treatments was observed for larval survival and dry weight of adult flies fed with cotton material and for adult dry weight and male wing size of flies fed with maize material. In contrast, flies fed with *Bt* cotton revealed a higher female wing size than those fed with non-*Bt* cotton when plants were infested with caterpillars (Fig. 1) and this effect could be attributed to the third trial (Supplemental Table S2). In this trial, rearing on infested *Bt* cotton also resulted in higher male wing size and shorter developmental time than on infested non-*Bt* cotton. No such differences were evident in the other trials as well as in the uninfested cotton treatments. Lower survival of *D. melanogaster* larvae feeding on *Bt* maize compared to non-*Bt* maize was observed in the treatments where plants were not infested with caterpillars (Fig. 1). This effect was solely attributed to the first of the three trials (trial 1), where larval survival on *Bt* maize was only 38% compared to 92% on non-*Bt* maize (Supplemental Table S2). In the other two trials, no such effect was observed and larval survival in the treatments with uninfested plants ranged between 74 and 92% independent from the *Bt* status. There was also no significant difference in survival when *D. melanogaster* fed *Bt* or non-*Bt* maize infested with caterpillars. In addition, trial 1 revealed longer development time for fly larvae reared on uninfested *Bt* maize than on uninfested non-*Bt* maize. When all trials were analyzed together, however, no difference between *D. melanogaster* fed with uninfested *Bt* and non-*Bt* maize was evident, but flies fed with infested *Bt* maize showed a shorter developmental time than those fed with infested non-*Bt* maize. Female wing size of flies reared on uninfested *Bt* maize was lower than on uninfested non-Bt maize, while no effect was evident for flies fed with infested *Bt* or non-*Bt* maize.

Purified Cry proteins showed no adverse effect on lethal or sublethal parameters when all three trials were analyzed in a combined analysis. However, trial 1 resulted in lower developmental time for Cry1B and higher female dry weight for Cry1Ac compared to the untreated control, while no effects were evident in the other trials. Cryolite served as a positive control in the *D. melanogaster* assays. As expected, this compound resulted generally in concentration-dependent adverse effects on survival, developmental time, dry weight, and partly also on wing size.

Overall, some differences between *Bt* and non-*Bt* treatments in individual trials were manifested as significant for trial, trial × treatment, and treatment in the overall analysis or in the analyses for individual trials. Those differences, however, appeared to be randomly positive or negative when *Bt* and non-*Bt* treatments were compared, not consistent over the three trials, and/or not consistent over infested and uninfested plant treatments. This highlights the value of conducting several independent trials over time to identify and confirm potential trends. In summary, we conclude that our experiments did not provide evidence that *D. melanogaster* is adversely affected by *Bt* maize (SmartStax), *Bt* cotton (Bollgard II), or the tested purified Cry proteins.

To our knowledge, the only published study investigating the effects of *Bt* plants on *D. melanogaster* is by Knecht and Nentwig^10^, who did not find evidence for adverse effects of *Bt* maize expressing Cry1Ab or Cry3Bb1 in feeding trials spanning 4 generations. A second saprophagous species tested in this study, i.e. *Megaselia scalaris* (Diptera: Phoridae) was also not affected by the *Bt* maize. Similar to our study, however, some inconsistent significances in one or the other generation had been observed.

Some Cry proteins have been shown to affect other groups within the Diptera. For example, Culicidae are sensitive to Cry2A^11^. Our current knowledge about the impact of specific Cry proteins on Diptera is limited since many previous studies used *Bt* formulations or serotypes that contained numerous Cry proteins and other insecticidal factors^19–22^.

### Cry-protein uptake

The presence of Cry protein in larvae and the fact that the larval gut turned green confirmed that *D. melanogaster* larvae ingest Cry protein when feeding on artificial diet containing 30% plant material. However, Cry protein concentrations measured in the fly larvae were low, ranging from 0.1 to 2.4% of the concentrations in the leaf powder, while Cry1Ac in larvae feeding on cotton material was not detectable.

Furthermore, the ELISA analyses revealed that adult flies did not contain measurable amounts of any Cry protein following cotton and maize larval exposure (except for 3 samples containing traces of Cry34Ab1). We therefore conclude that the Cry proteins were not taken up into the larval bodies and that gut contents containing Cry protein were excreted prior to pupation. Our results are in accordance with those by Knecht and Nentwig^10^ who found low Cry protein concentration in *D. melanogaster* and *M. scalaris* larvae, but no detectable amounts in adult flies. Field studies where adult Diptera from different functional groups and families were collected in *Bt* maize, cotton and soybean fields report mixed results. While in the majority of samples no Cry protein could be detected^23–25^, some exceptions were reported. This includes *Oscinella frit* flies (Diptera: Chloropidae) collected in *Bt* maize that contained 17 µg Cry3Bb1 per g DW^24^ and low levels (0.06–0.07 µg Cry1Ac per g DW) in an unidentified saprophagous species belonging to the family of Neriidae collected in Bt soybean^25^.

### Effects of herbivore infestation

When cotton plants were infested with *H. virescens* caterpillars, natural plant defence was activated, which was evidenced by the elevated terpenoid concentrations in leaf samples. Terpenoids are known to be toxic to a wide range of insect orders including Diptera^13, 26–31^. Terpenoid concentrations in newly developed leaves more than doubled following caterpillar feeding on the lower leaves compared with uninfested plants. Induction was equally observed for *Bt* and non-*Bt* cotton. Because late L2 or early L3 *H. virescens* were used to induce the plants, the larvae were less susceptible to *Bt* cotton than neonates, and feeding damage was observed, albeit at a lower level than on non-*Bt* cotton.

Feeding *D. melanogaster* with induced cotton plant material resulted in a significantly prolonged developmental time compared to flies fed undamaged plant material. Similarly, dry weight and wing size of the adult flies tended to be lower, even though only some comparisons were significant. In maize, there was no indication that caterpillar infestation influenced *D. melanogaster* performance. There are reports that also maize has some inducible defence substances, such as DIMBOA, which might play a role in pathogen or insect defence, for example against Lepidoptera and Hemiptera^32^, although effects on insects would likely be less evident for maize than for cotton.

### Quality control of the assay system

The assay system employed has previously been used to determine potential effects of a range of gut-active insecticidal compounds on *D. melanogaster* ^16^. Cryolite, an inorganic pesticide that was used as a positive control, resulted in lethal and sublethal responses as previously observed^16^. Survival in the diet-only control treatment (negative control) was well above 80% except for one assay, which was discarded for further analyses. This shows the importance of positive and negative control treatments to ensure reliable assay performance. We demonstrated that the assay system is also suitable to test the effects of plant material on *D. melanogaster*, in addition to purified insecticidal proteins. Mixing one third of maize or cotton leaf powder into the artificial diet did not result in a reduction of *D. melanogaster* performance.

ELISA measurements revealed that the process of mixing the purified Cry proteins into the artificial diet, lyophilizing the diet, and extracting the proteins from the diet using PBST, resulted in estimates between 31% (Cry1Ab) and 79% (Cry1F) of the originally applied Cry protein concentration. When the formulated diet was aged for five days under the environmental conditions used for the bioassay, Cry protein concentrations decreased further by 32% (Cry2Aa) to 84% (Cry1F). This indicates that Cry proteins may have adsorbed to components of the artificial diet, may have been degraded and/ or became deactivated by compounds present in the diet. The loss of Cry protein after mixing into the diet as well as the loss during incubation in the assay differs among the Cry proteins. While Cry1F concentrations were high in the fresh diet, most of the Cry protein was not detected after a 5-day period. In contrast, Cry2Aa concentrations in the fresh diet were low, but further loss during the assay was limited. Knecht and Nentwig^10^ reported a comparable decline in Cry1Ab and Cry3Bb1 concentrations in artificial diets during a one week exposure period.

Because of the demonstrated loss of measurable Cry proteins, and because it is possible that ELISA may detect the presence of inactivated Cry proteins, it is important to confirm the biological activity of the Cry proteins in an artificial diet assay using sensitive insects. In the present study, we were able to confirm the biological activity of Cry1Ab, Cry1Ac, Cry1F, and Cry2Aa in the artificial diet based on the known sensitivity of *H. virescens* neonates to those Cry proteins^33–35^. No clear biological activity was observed for Cry1B and Cry1C, but there is also no indication from the literature that those Cry proteins are active against *H. virescens*. As expected, formulated diet treatments aged for 5 days showed lower bioactivity than freshly prepared diet, where treatment effects were more readily detected for sublethal endpoints than lethal ones. Overall, the sensitive insect bioassays confirmed that our artificial diet system delivered biologically active Cry proteins to *D. melanogaster* larvae via oral consumption, even though a part of the Cry proteins were not measurable after mixing into the diet and concentrations in the artificial diet decreased over time.

## Conclusions

The current study provides evidence that there are no consistent adverse effects of a range of purified Cry1 and Cry2 proteins or *Bt* plants producing multiple Cry proteins on *D. melanogaster*, a decomposer Diptera. In contrast, caterpillar infestation of cotton leaves resulted in longer development time and smaller wing size of *D. melanogaster* fed this material compared to flies fed uninfested leaves, while no such effects were obvious for maize.

## Electronic supplementary material

Supplemental information

Supplementary Dataset 1
