# Supplementary material for: Effects of purified or plant-produced Cry proteins on Drosophila melanogaster (Diptera: Drosophilidae) larvae
Source: Sci Rep. 2017 Sep 11;7:11172. doi: 10.1038/s41598-017-10801-4 (PMC5593937; doi:10.1038/s41598-017-10801-4)
Supplement: Supplementary file 1 — Supplemental information [file 41598_2017_10801_MOESM1_ESM.pdf]

Supplemental information

Effects of purified or plant-produced Cry proteins on *Drosophila melanogaster* (Diptera: Drosophilidae) larvae

Simone Haller, Jörg Romeis, Michael Meissle

Scientific Reports

Supplemental Table S1 Statistics of the plant material assay with *Drosophila melanogaster*. The effects of treatments were tested with a generalized linear model using Chi-square statistics for the survival data (binomial distribution) and F-statistics for the sublethal measurement endpoints (quasipoisson distribution). When treatment was significant, means were compared using Tukey's post-hoc test (see Fig. 1 in the main manuscript). When treatment × trial interactions were significant, the analysis was repeated for each trial separately (see footnotes). The results of Tukey's post-hoc test for those analyses are provided in Table S2.

		**Generalized linear model**								
		Treatment			Trial			Treatment × trial interaction		
**Parameter**	**Treatment**	df	*X* / *F*	*P*	df	*X* / *F*	*P*	df	*X* / *F*	*P*
Survival	Cryolite	2, 197	240.65	< 0.001	2, 195	218.38	n.s.	4, 191	218.08	n.s.
	Cotton	3, 250	231.02	n.s.	2, 248	230.16	n.s.	6, 242	233.14	n.s.
	Maize	3, 252	253.32	0.002	2, 250	242.82	n.s.	6, 244	253.59	0.019[1]
Developmental time	Cryolite	2, 101	6.47	0.002	2, 101	6.64	0.002	4, 101	0.75	n.s.
	Cotton	3, 197	10.96	< 0.001	2, 197	28.90	< 0.001	6, 197	2.24	0.029[2]
	Maize	3, 190	3.81	0.011	2, 190	37.31	< 0.001	6, 190	3.33	0.004[3]
Female dry weight	Cryolite	1, 21	7.88	0.011	2, 21	5.98	0.009	2, 21	1.90	n.s.
	Cotton	3, 80	1.90	n.s.	2, 80	1.49	n.s.	6, 80	1.41	n.s.
	Maize	8, 83	1.21	n.s.	2, 83	5.20	0.007	6, 83	1.10	n.s.
Male dry weight	Cryolite	2, 66	18.11	< 0.001	2, 66	16.44	< 0.001	4, 66	0.89	n.s.
	Cotton	3, 82	1.08	n.s.	2, 82	2.78	n.s.	6, 82	1.48	n.s.
	Maize	3, 85	2.59	n.s.	2, 85	10.69	< 0.001	6, 85	0.08	n.s.
Females wing size	Cryolite	1, 18	0.04	n.s.	2, 18	0.04	n.s.	2, 18	0.85	n.s.
	Cotton	3, 71	12.72	< 0.001	2, 71	1.30	n.s.	6, 71	5.41	< 0.001[4]
	Maize	3, 73	3.38	0.023	2, 73	6.25	0.003	6, 73	0.61	n.s.
Male wing size	Cryolite	2, 52	4.92	0.011	2, 52	4.14	0.021	4, 52	1.93	n.s.
	Cotton	3, 74	8.50	< 0.001	2, 74	1.83	n.s.	6, 74	3.51	0.004[5]
	Maize	3, 77	0.24	n.s.	2, 77	3.12	0.050	6, 77	1.96	n.s.

[1] Trial 1: $X_{71,3} = 88.99$, p = 0.002
Trial 2: $X_{86,3} = 79.80$, n.s.
Trial 3: $X_{87,3} = 95.25$, n.s.

[2] Trial 1: $F_{66,3} = 3.03$, p = 0.035
Trial 2: $F_{67,3} = 6.18$, p < 0.001
Trial 3: $F_{64,3} = 7.16$, p < 0.001

[3] Trial 1: $F_{51,3} = 9.00$, p < 0.001
Trial 2: $F_{71,3} = 0.52$, n.s.
Trial 3: $F_{68,3} = 2.53$, n.s.

[4] Trial 1: $F_{18,3} = 0.21$, n.s.
Trial 2: $F_{28,3} = 15.34$, p < 0.001
Trial 3: $F_{25,3} = 7.23$, p < 0.001

[5] Trial 1: $F_{25,3} = 0.05$, n.s.
Trial 2: $F_{25,3} = 6.45$, p = 0.002
Trial 3: $F_{24,3} = 7.28$, p < 0.001

Supplemental Table S2 Effects of *Bt* cotton and *Bt* maize diet, caterpillar-infested or not infested, on survival, developmental time, dry weight and wing size of *Drosophila melanogaster*. Values are means ± SE; n = 24 per treatment and trial at the start of the experiment. As positive control, cryolite at 0.004 % and 0.04 % were used to assess sublethal and lethal effects, respectively. If significant trial × treatment interactions occurred in the overall analysis including all trials, each trial was analyzed separately (see Table S1). Significant treatment effects in the individual trials were further explored with Tukey's post-hoc tests. Those results are given in letters next to the respective parameter in this table.

Treatment	Trial	Survival [%]	Developmental time [d]	Female dry weight [µg]	Male dry weight [µg]	Female wing size [µm]	Male wing size [µm]
Negative control	1	76.19	9.44 ± 0.16	215.67 ± 47.68	137.08 ± 8.22	1969.00	1735.29 ± 9.65
Cryolite 0.004 %	1	54.17	9.38 ± 0.14	93.00	110.80 ± 7.75	2063.00	1769.57 ± 33.06
Cryolite 0.04 %	1	8.70	10.50 ± 0.50	-	83.00 ± 15.00	-	1720.50 ± 45.50
Cotton	1	73.68	9.57 ± 0.14 **ab**	193.29 ± 14.67	169.83 ± 33.56	1946.29 ± 34.99	1743.20 ± 26.57
Cotton infested	1	91.67	9.82 ± 0.16 **ab**	220.80 ± 82.91	137.87 ± 10.45	1926.25 ± 35.24	1745.17 ± 32.13
Bt cotton	1	76.19	9.31 ± 0.15 **a**	228.14 ± 16.80	176.44 ± 16.79	1925.67 ± 52.08	1738.33 ± 23.98
Bt cotton infested	1	85.71	9.94 ± 0.17 **b**	209.33 ± 16.09	153.63 ± 19.67	1966.00 ± 19.70	1727.83 ± 34.53
Maize	1	91.91 **b**	8.90 ± 0.12 **a**	193.60 ± 35.75	132.46 ± 8.77	1947.00 ± 4.04	1778.75 ± 37.1
Maize infested	1	80.00 **ab**	9.63 ± 0.13 **bc**	147.00	129.57 ± 8.84	1906.00	1710.17 ± 18.21
Bt maize	1	37.50 **a**	10.14 ± 0.40 **c**	189.00 ± 80.00	160.00 ± 40.45	1777.00	1724.25 ± 60.38
Bt maize infested	1	64.71 **ab**	9.00 ± 0.19 **ab**	181.25 ± 28.03	142.29 ± 21.68	1962.33 ± 13.32	1811.17 ± 27.90
Negative control	2	75.00	9.13 ± 0.43	262.33 ± 16.84	196.00 ± 11.79	2040.57 ± 28.15	1811.17 ± 27.90
Cryolite 0.004 %	2	56.52	9.08 ± 0.35	205.33 ± 22.17	154.71 ± 12.73	1953.00 ± 33.50	1760.83 ± 27.35
Cryolite 0.04 %	2	21.74	10.40 ± 0.75	205.00 ± 45.90	97.50 ± 4.50	1988.67 ± 48.33	1541.00
Cotton	2	91.30	8.71 ± 0.12 **a**	283.40 ± 71.55	187.79 ± 13.79	2006.40 ± 25.85 **b**	1774.93 ± 21.40 **b**
Cotton infested	2	84.21	9.50 ± 0.27 **bc**	199.50 ± 15.80	205.00 ± 5.49	1800.13 ± 34.96 **a**	1621.20 ± 37.66 **a**
Bt cotton	2	85.00	8.94 ± 0.10 **ab**	244.60 ± 19.04	175.00 ± 19.61	2014.89 ± 23.41 **b**	1779.67 ± 28.18 **b**
Bt cotton infested	2	80.95	9.82 ± 0.32 **c**	210.50 ± 21.22	143.50 ± 31.62	1809.40 ± 29.31 **a**	1610.25 ± 76.35 **a**
Maize	2	77.27	8.76 ± 0.25	291.25 ± 25.04	166.43 ± 9.54	2061.00 ± 15.10	1742.63 ± 32.27
Maize infested	2	90.91	8.62 ± 0.13	215.78 ± 15.23	156.27 ± 12.91	1987.56 ± 24.12	1836.75 ± 45.82
Bt maize	2	91.67	8.64 ± 0.10	197.56 ± 15.85	201.80 ± 24.08	1978.50 ± 23.95	1765.40 ± 54.43
Bt maize infested	2	76.19	8.40 ± 0.34	236.38 ± 28.13	184.75 ± 20.36	2017.50 ± 14.88	1816.17 ± 29.02
Negative control	3	89.47	8.35 ± 0.12	281.67 ± 12.99	208.23 ± 10.30	2020.33 ± 10.91	1838.33 ± 21.99
Cryolite 0.004 %	3	75.00	8.89 ± 0.15	261.25 ± 20.14	159.18 ± 13.59	1982.67 ± 54.20	1783.91 ± 34.81
Cryolite 0.04 %	3	41.67	9.40 ± 0.22	-	147.80 ± 11.55	-	1758.00 ± 31.08
Cotton	3	85.71	8.24 ± 0.11 **a**	292.10 ± 20.34	201.57 ± 7.65	2067.75 ± 15.35 **b**	1790.14 ± 10.32 **b**
Cotton infested	3	72.73	9.25 ± 0.11 **b**	180.43 ± 14.23	164.50 ± 11.91	1758.17 ± 39.65 **a**	1602.40 ± 49.68 **a**
Bt cotton	3	82.61	8.63 ± 0.17 **a**	249.33 ± 20.52	183.14 ± 5.91	1964.86 ± 59.93 **b**	1771.75 ± 15.89 **b**
Bt cotton infested	3	80.00	8.63 ± 0.20 **a**	280.80 ± 37.94	196.43 ± 13.24	1974.75 ± 21.43 **b**	1753.13 ± 20.68 **b**
Maize	3	76.19	8.50 ± 0.13	259.25 ± 28.14	188.92 ± 6.39	2038.33 ± 14.52	1773.30 ± 14.72
Maize infested	3	83.33	8.45 ± 0.15	245.31 ± 10.44	173.50 ± 20.20	1974.25 ± 22.67	1698.60 ± 36.14
Bt maize	3	73.91	8.18 ± 0.13	258.82 ± 12.90	211.00 ± 22.72	1988.80 ± 30.58	1729.33 ± 23.98
Bt maize infested	3	82.61	8.11 ± 0.07	240.21 ± 10.95	199.20 ± 23.61	1992.85 ± 13.67	1769.60 ± 36.62

Supplemental Table S3 Statistics of the purified Cry protein assay with *Drosophila melanogaster*. The effects of treatments were tested with a generalized linear model using Chi-square statistics for the survival data (binomial distribution) and F-statistics for the sublethal measurement endpoints (quasipoisson distribution). When treatment was significant, means were compared to the untreated control diet using Dunnett's post hoc test (see Fig. 2 in the main manuscript). When treatment × trial interactions were significant, the analysis was repeated for each trial separately (see footnotes). The results of Dunnett's post-hoc test for those analyses are provided in Table S4.

		**Generalized linear model**								
		Treatment			Trial			Treatment × trial interaction		
**Parameter**	**Treatment**	df	*X / F*	*P*	df	*X / F*	*P*	df	*X / F*	*P*
Survival	Cryolite	2, 224	314.93	< 0.001	2, 224	254.37	0.004	4, 224	239.85	n.s.
	Cry proteins	6, 421	415.78	n.s.	2, 421	432.34	< 0.001	12, 421	403.33	n.s.
Developmental time	Cryolite	2, 132	111.77	< 0.001	2, 132	1.45	n.s.	4, 132	1.60	n.s.
	Cry proteins	6, 332	1.18	n.s.	2, 332	37.78	< 0.001	12, 332	3.30	< 0.001[1]
Female dry weight	Cryolite	1, 55	2.19	n.s.	2, 55	0.98	n.s.	2, 55	3.12	n.s.
	Cry proteins	6, 136	0.36	n.s.	2, 136	1.81	n.s.	12, 136	2.21	0.014[2]
Male dry weight	Cryolite	2, 58	11.05	< 0.001	2, 58	7.18	0.002	4, 58	2.98	0.026[3]
	Cry proteins	6, 117	0.66	n.s.	2, 117	8.97	< 0.001	12, 117	2.16	0.018[4]
Female wing size	Cryolite	1, 43	1.21	n.s.	2, 43	0.91	n.s.	2, x	0.43	n.s.
	Cry proteins	6, 121	1.77	n.s.	2, 121	7.12	0.001	12, 121	1.20	n.s.
Male wing size	Cryolite	2, 52	2.87	n.s.	2, 52	1.57	n.s.	4, 52	3.52	0.013[5]
	Cry proteins	6, 118	0.62	n.s.	2, 118	3.89	0.023	12, 118	0.53	n.s.

[1] Trial 1: $F_{86,6} = 3.90$, p = 0.002
Trial 2: $F_{127,6} = 1.97$, n.s.
Trial 3: $F_{119,6} = 1.74$, n.s.

[2] Trial 1: $F_{41,6} = 2.74$, p = 0.025
Trial 2: $F_{41,6} = 0.42$, n.s.
Trial 3: $F_{54,6} = 1.98$, n.s.

[3] Trial 1: $F_{16,2} = 3.11$, n.s.
Trial 2: $F_{18,2} = 3.80$, p = 0.042
Trial 3: $F_{24,2} = 13.88$, p < 0.001

[4] Trial 1: $F_{24,6} = 2.32$, n.s.
Trial 2: $F_{41,6} = 2.08$, n.s.
Trial 3: $F_{53,6} = 0.78$, n.s.

[5] Trial 1: $F_{15,2} = 0.96$, n.s.
Trial 2: $F_{17,2} = 0.68$, n.s.
Trial 3: $F_{20,2} = 6.70$, p = 0.006

Supplemental Table S4 Effects of different Cry proteins at a concentration of 0.01 % (w/w) on survival, developmental time, dry weight and wing size of *Drosophila melanogaster*. Values are means ± SE; n = 21 per treatment and trial at the start of the experiment. As positive control, cryolite at 0.004 % and 0.04 % were used to assess sublethal and lethal effects, respectively. If significant trial × treatment interactions occurred in the overall analysis including all trials, each trial was analyzed separately (see Table S3). Significant treatment effects in the individual trials were further explored with Dunnett's post-hoc tests. Those results are given in asterisks (* p<0.05, ** p<0.01, *** p<0.005) next to the respective parameter in this table.

**Treatment**	**Trial**	**Survival [%]**	**Developmental time [d]**	**Female dry weight [µg]**	**Male dry weight [µg]**	**Female wing size [µm]**	**Male wing size [µm]**
Negative control	1	82.50	8.50 ± 0.14	231.00 ± 16.42	156.82 ± 14.14	2059.90 ± 31.51	1773.91 ± 24.14
Cryolite 0.004 %	1	33.33	9.29 ± 0.18	217.00	118.67 ± 12.07	2048.00	1775.00 ± 31.30
Cryolite 0.04 %	1	14.29	11.33 ± 0.33	63.00	92.00 ± 26.00	1915.00	1858.50 ± 79.50
Cry1Ab	1	80.95	8.71 ± 0.13	269.20 ± 16.69	181.00 ± 7.00	2066.56 ± 26.53	1843.25 ± 46.70
Cry1Ac	1	68.42	8.46 ± 0.18	364.78 ± 48.59 **	207.00 ± 16.00	2073.11 ± 20.61	1759.33 ± 46.06
Cry1B	1	66.66	7.92 ± 0.08 **	264.50 ± 43.12	198.00 ± 10.26	2090.75 ± 20.47	1833.80 ± 33.10
Cry1C	1	80.00	8.33 ± 0.17	325.25 ± 27.49	154.75 ± 9.53	2041.50 ± 22.50	1758.80 ± 80.00
Cry1F	1	25.00 **	8.50 ± 0.22	302.00	150.00 ± 5.00	2030.00	1830.00 ± 60.70
Cry2Aa	1	76.19	8.73 ± 0.14	235.33 ± 34.87	117.25 ± 21.46	1927.00	1772.00
Negative control	2	80.03	9.10 ± 0.16	290.80 ± 17.76	217.67 ± 11.64	2041.50 ± 16.78	1843.67 ± 30.49
Cryolite 0.004 %	2	61.90	9.17 ± 0.11	191.57 ± 32.37	134.67 ± 18.00 *	2006.00 ± 21.02	1796.40 ± 23.26
Cryolite 0.04 %	2	21.05	11.75 ± 0.48		160.33 ± 38.69	-	1793.00 ± 44.86
Cry1Ab	2	89.47	9.00	276.86 ± 29.07	199.40 ± 9.68	2046.20 ± 48.86	1788.89 ± 17.57
Cry1Ac	2	90.00	9.00	225.25 ± 67.47	140.80 ± 15.90	2060.57 ± 24.97	1792.00 ± 20.49
Cry1B	2	88.88	8.88 ± 0.09	244.00 ± 33.05	157.71 ± 19.07	2079.63 ± 19.59	1850.57 ± 55.73
Cry1C	2	95.25	9.47 ± 0.29	265.83 ± 45.98	191.67 ± 29.85	2025.62 ± 22.28	1781.00 ± 30.51
Cry1F	2	88.25	9.08 ± 0.08	262.14 ± 30.30	223.00 ± 37.72	2061.33 ± 17.88	1806.86 ± 27.01
Cry2Aa	2	85.71	9.00	252.60 ± 32.92	171.25 ± 39.59	2058.43 ± 27.02	1788.00 ± 27.01
Negative control	3	91.82	8.85 ± 0.15	261.13 ± 16.97	231.35 ± 11.07	2153.27 ± 74.54	1891.67 ± 46.76
Cryolite 0.004 %	3	71.43	9.07 ± 0.12	263.33 ± 17.15	237.20 ± 33.02	2018.50 ± 17.14	1725.67 ± 53.18
Cryolite 0.04 %	3	38.09	11.57 ± 0.37	196.50 ± 77.50	113.80 ± 15.29 ***	1913.50 ± 184.50	1614.60 ± 36.16 **
Cry1Ab	3	81.25	9.00	312.43 ± 33.15	195.33 ± 16.79	2304.00 ± 87.64	1896.00 ± 97.90
Cry1Ac	3	90.00	9.12 ± 0.08	264.85 ± 19.23	204.00 ± 30.32	2085.92 ± 33.14	1925.50 ± 60.20
Cry1B	3	84.21	9.18 ± 0.21	294.75 ± 12.54	202.00 ± 9.90	2247.20 ± 114.37	1878.33 ± 74.51
Cry1C	3	83.33	8.73 ± 0.12	273.00 ± 14.22	230.11 ± 23.22	2066.75 ± 22.87	1835.14 ± 39.26
Cry1F	3	88.89	8.86 ± 0.09	273.60 ± 18.20	208.60 ± 15.03	2110.25 ± 9.20	1819.56 ± 13.84
Cry2Aa	3	68.42	8.85 ± 0.10	356.67 ± 37.23	226.43 ± 21.64	2044.00 ± 15.82	1805.14 ± 13.64

Supplemental Table S5 Effects of *Drosophila melanogaster* diet containing different Cry proteins, on survival and fresh weight (mean ± SE) of *Heliothis virescens*. The treated *D. melanogaster* diet, either freshly prepared ("fresh") or incubated for 5 days ("old"), was lyophilized, pulverized, and mixed into artificial diet for *H. virescens*. n = 20 per treatment and per trial. The experiment was conducted in two parts (A and B) and in two trials for each part.

Treatment	Trial	Part	Survival [%]	Fresh weight [µg]	95 % confidence interval
stonefly A	1	A	95	23.51 ± 2.11	19.49 - 27.52
D. melanogaster control diet 10 mg, fresh	1	A	95	17.64 ± 2.67	12.56 - 22.72
D. melanogaster control diet 10 mg, old	1	A	95	16.96 ± 3.00	11.23 - 22.69
Cry1Ab 10 mg, fresh	1	A	80	2.32 ± 0.40	1.55 - 3.08
Cry1Ab 10 mg, old	1	A	80	8.56 ± 1.63	5.46 - 11.65
Cry1Ac 10 mg, fresh	1	A	35	0.49 ± 0.09	0.33 - 0.65
Cry1Ac 10 mg, old	1	A	85	2.33 ± 0.45	1.47 - 3.19
Cry2Aa 10 mg, fresh	1	A	85	3.84 ± 0.97	1.99 - 5.68
Cry2Aa 10 mg, old	1	A	55	4.78 ± 1.89	1.25 - 8.31
stonefly B	1	B	81	14.17 ± 2.57	9.33 - 19.02
D. melanogaster control diet 100 mg, fresh	1	B	80	29.79 ± 11.83	7.59 - 51.99
D. melanogaster control diet 100 mg, old	1	B	88	14.03 ± 2.87	8.59 - 19.46
D. melanogaster control diet 1000 mg, fresh	1	B	84	21.86 ± 5.45	11.55 - 32.16
D. melanogaster control diet 1000 mg, old	1	B	78	28.48 ± 10.66	8.34 - 48.62
Cry1B 1000 mg, fresh	1	B	53	7.23 ± 2.03	3.48 - 10.98
Cry1B 1000 mg, old	1	B	53	17.78 ± 7.57	3.70 - 31.86
Cry1C 1000 mg, fresh	1	B	45	11.76 ± 3.95	4.46 - 19.05
Cry1C 1000 mg, old	1	B	65	15.08 ± 5.95	4.60 - 27.00
Cry2F 100 mg, fresh	1	B	5	4.30	-
Cry2F 100 mg, old	1	B	75	3.53 ± 0.85	1.91 - 5.14
stonefly A	2	A	84	19.62 ± 4.40	27.97 - 11.28
D. melanogaster control diet 10 mg, fresh	2	A	82	22.70 ± 8.27	7.09 - 38.32
D. melanogaster control diet 10 mg, old	2	A	82	9.60 ± 1.70	6.39 - 12.80
Cry1Ab 10 mg, fresh	2	A	5	0.55	-
Cry1Ab 10 mg, old	2	A	65	5.97 ± 0.71	4.62 - 7.31
Cry1Ac 10 mg, fresh	2	A	35	1.06 ± 0.32	0.48 - 1.63
Cry1Ac 10 mg, old	2	A	50	2.34 ± 0.69	1.05 - 3.62
Cry2Aa 10 mg, fresh	2	A	50	7.67 ± 2.50	3.05 - 12.28
Cry2Aa 10 mg, old	2	A	35	3.17 ± 0.66	1.98 - 4.36
stonefly B	2	B	90	50.50 ± 8.07	35.13 - 65.87
D. melanogaster control diet 100 mg, fresh	2	B	89	61.08 ± 10.41	41.30 - 80.87
D. melanogaster control diet 100 mg, old	2	B	89	57.89 ± 10.72	37.50 - 78.27
D. melanogaster control diet 1000 mg, fresh	2	B	95	79.49 ± 9.02	62.34 - 96.64
D. melanogaster control diet 1000 mg, old	2	B	95	98.70 ± 8.19	83.08 - 114.31
Cry1B 1000 mg, fresh	2	B	100	16.13 ± 2.85	10.69 - 21.56
Cry1B 1000 mg, old	2	B	94	24.76 ± 3.10	18.88 - 30.65
Cry1C 1000 mg, fresh	2	B	89	14.64 ± 2.66	9.59 - 19.70
Cry1C 1000 mg, old	2	B	76	21.51 ± 1.98	17.76 - 25.26
Cry2F 100 mg, fresh	2	B	35	2.46 ± 0.51	1.54 - 3.38
Cry2F 100 mg, old	2	B	75	8.42 ± 1.36	5.84 - 10.99

Supplemental Table S6 Cry protein concentrations measured in individual *Drosophila melanogaster* larvae and adult flies, reared on *Bt* plant material (Bollgard II® cotton and SmartStax™ maize). Proteins not analyzed for a particular sample are indicated with n.e.

Treatment	Sample	***Bt* concentration [µg/g DW]** Cry1Ac / Cry1A.105	Cry1F	Cry2Ab	Cry3Bb1	Cry34Ab1
Larvae on *Bt* cotton	1	<0.016	n.e.	0.032	n.e.	n.e.
	2	<0.015	n.e.	0.041	n.e.	n.e.
	3	<0.011	n.e.	0.027	n.e.	n.e.
	4	<0.012	n.e.	0.038	n.e.	n.e.
	5	<0.017	n.e.	0.052	n.e.	n.e.
	6	<0.016	n.e.	0.143	n.e.	n.e.
	7	<0.013	n.e.	0.052	n.e.	n.e.
	8	<0.008	n.e.	0.241	n.e.	n.e.
	9	<0.014	n.e.	0.110	n.e.	n.e.
	10	<0.016	n.e.	0.173	n.e.	n.e.
Larvae on *Bt* maize	1	0.212	0.058	0.018	0.030	0.129
	2	0.253	0.041	0.034	0.033	0.304
	3	0.687	0.085	0.115	0.111	0.396
	4	0.257	0.103	0.060	0.065	0.061
	5	<0.171	0.018	0.027	0.034	0.053
	6	0.269	0.045	0.038	0.037	0.140
	7	1.606	0.057	0.042	0.099	0.487
	8	0.981	0.047	0.038	0.058	0.225
	9	0.630	0.122	0.043	0.065	0.324
Adults on *Bt* cotton	1	<0.023	n.e.	<0.008	n.e.	n.e.
	2	<0.018	n.e.	<0.006	n.e.	n.e.
	3	<0.019	n.e.	<0.006	n.e.	n.e.
	4	<0.010	n.e.	<0.003	n.e.	n.e.
	5	<0.026	n.e.	<0.009	n.e.	n.e.
	6	<0.010	n.e.	<0.003	n.e.	n.e.
	7	<0.012	n.e.	<0.004	n.e.	n.e.
	8	<0.015	n.e.	<0.004	n.e.	n.e.
	9	<0.008	n.e.	<0.003	n.e.	n.e.
Adults on *Bt* maize	1	<0.273	<0.010	<0.009	<0.007	<0.003
	2	<0.293	<0.011	<0.008	<0.008	0.005
	3	<0.128	<0.005	<0.003	<0.003	<0.001
	4	<0.288	<0.011	<0.008	<0.008	0.022
	5	<0.173	<0.007	<0.006	<0.005	<0.002
	6	<0.131	<0.005	<0.004	<0.003	<0.001
	7	<0.171	<0.006	<0.006	<0.004	<0.002
	8	<0.175	<0.007	<0.006	<0.005	0.002

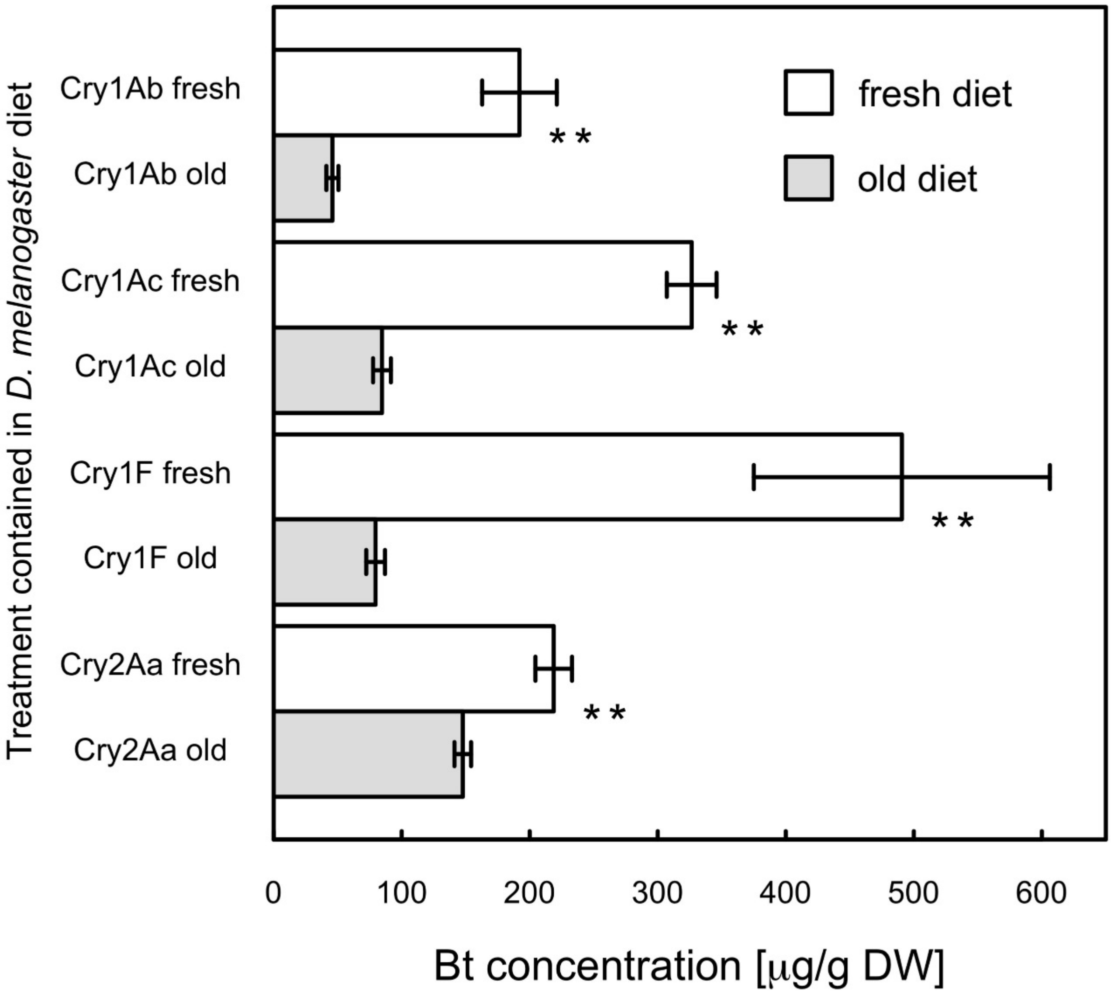

Supplemental Figure 1 Cry protein concentrations in *Drosophila melanogaster* diet (freshly prepared and after five days incubation). Values are means ± SE of five subsamples taken from one pool of lyophilized and pulverized artificial diet. Asterisks denote a significant difference between fresh and 5 days old diet (Mann-Whitney Wilcoxon test, $p<0.01$).

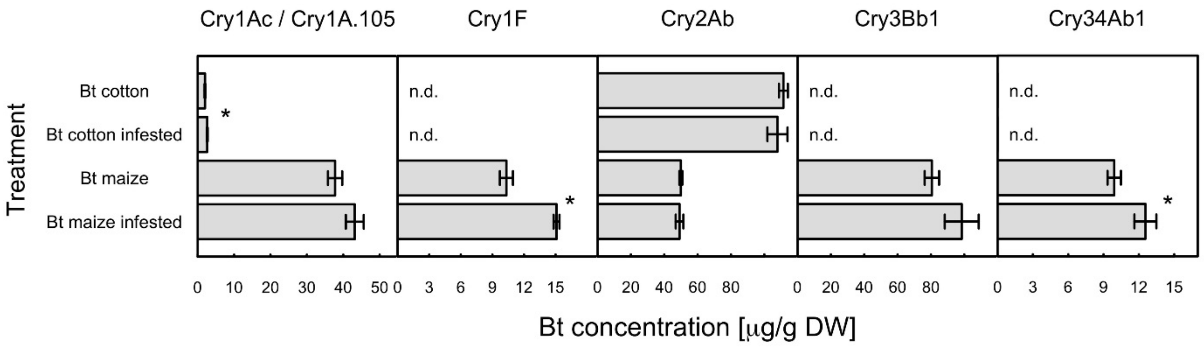

Supplemental Figure 2 Cry protein concentrations in milled leaves of *Bt* cotton (Bollgard II®) and maize (SmartStax™), from caterpillar-infested or uninfested plants. Values are means ± SE of five subsamples taken from one pool of lyophilized and pulverized leaf material. n.d. indicates that the respective Cry protein was not determined. Asterisks denote a significant difference between uninfested and infested plants (Mann-Whitney Wilcoxon test, $p<0.05$).
